# Mechanisms of Improved Aortic Stiffness by Arotinolol in Spontaneously Hypertensive Rats

**DOI:** 10.1371/journal.pone.0088722

**Published:** 2014-02-12

**Authors:** Wugang Zhou, Mona Hong, Ke Zhang, Dongrui Chen, Weiqing Han, Weili Shen, Dingliang Zhu, Pingjin Gao

**Affiliations:** Department of Hypertension, RuiJin Hospital, Shanghai Key Lab of Hypertension, Shanghai Institute of Hypertension, Shanghai Jiaotong University, School of Medicine, Shanghai, China; Idaho State University, United States of America

## Abstract

**Objectives:**

This study investigates the effects on aortic stiffness and vasodilation by arotinolol and the underlying mechanisms in spontaneously hypertensive rats (SHR).

**Methods:**

The vasodilations of rat aortas, renal and mesenteric arteries were evaluated by isometric force recording. Nitric oxide (NO) was measured in human aortic endothelial cells (HAECs) by fluorescent probes. Sixteen-week old SHRs were treated with metoprolol (200 mg·kg-1·d-1), arotinolol (30 mg·kg-1·d-1) for 8 weeks. Central arterial pressure (CAP) and pulse wave velocity (PWV) were evaluated via catheter pressure transducers. Collagen was assessed by immunohistochemistry and biochemistry assay, while endothelial nitric oxide synthase (eNOS) and eNOS phosphorylation (p-eNOS) of HAECs or aortas were analyzed by western blotting.

**Results:**

Arotinolol relaxed vascular rings and the relaxations were attenuated by Nω-nitro-L-arginine methyl ester (L-NAME, NO synthase inhibitor) and the absence of endothelium. Furthermore, arotinolol-induced relaxations were attenuated by 4-aminopyridine (4-AP, Kv channels blocker). Arotinolol produced more nitric oxide compared to metoprolol and increased the expression of p-eNOS in HAECs. These results indicated that arotinolol-induced vasodilation involves endothelium-derived NO and Kv channels. The treatement with arotinolol in 8 weeks, but not metoprolol, markedly decreased CAP and PWV. Biochemistry assay and immunohistochemistry showed that aortic collagen depositions in the arotinolol groups were reduced compared with SHRs with metoprolol. Moreover, eNOS phosphorylation was significantly increased in aortinolol-treated SHR compared with SHRs with metoprolol.

**Conclusions:**

Arotinolol improves arterial stiffness in SHR, which involved in increasing NO and decreasing collagen contents in large arteries.

## Introduction

Beta-blockers (β-blockers) are widely prescribed for the treatment of a variety of cardiovascular pathologies including hypertension, heart failure, primary treatment of myocardial infarction, secondary prevention of ischemic cardiac events as well as other non-cardiovascular diseases. [1] The first generation β-blockers, for example propranolol, are nonselective and block both β_1_- and β_2_-adrenoceptors. The second generation β-blockers, including metoprolol, are cardioselective and selectively block β1-adrenoreceptors. There are discrepancies concerning the effect of metoprolol, a secondary generation β-blocker, on blood pressure and arterial stiffness. Kosch et al reported that metoprolol decreased central blood pressure and decreased aortic pulse wave velocity in hypertensive patients. [2] In contrast, there are studies showing that metoprolol does not have effect on central systolic and diastolic blood pressures, central pulse pressure, as well as left ventricular wall thickness.[3] The third generation vasodilating β-blockers, such as nebivolol, are β_1_-adrenoreceptors specific and have additional ancillary properties including direct vasodilatory effect, and studies show that the vasodilatory effect of nebivolol involves endothelium-derived nitric oxide (NO). [4] Compared to classic β-antagonists, they promote a vasodilation through different mechanisms, which lead to a more favorable hemodynamic profile compared to non-vasodilating β-blockers. Studies show that the third generation of β-blockers have beneficial effect on blood vessels in experimental and human hypertension, [5], [6] which are closely related to their vasodilatory effects.

Arotinolol is a nonselective α/β-adrenergic receptor blocker lack of local anesthetic, membrane-stabilizing or intrinsic sympathomimetic properties, which means that arotinolol is a pure α/β-adrenergic receptor blocker and vasodilating β-blocker as well.[7], [8] Arotinolol obtains some advantages from these characteristics since the third-generation β-blocker, such as nebivolol, had no effect on α-adrenergic receptor. Clinically, this drug has been extensively used for the treatment of hypertension. However, it remains unknown whether it has beneficial effect on arterial stiffness and it has few researches on whether vasodilatory effects of arotinolol get involved in endothelial functions.

It has been well documented that endothelial function was impaired and arterial stiffness was significantly higher in spontaneously hypertensive rats (SHR) compared with normotensive Wista Kyoto (WKY) rats. [9] In the present study, we investigated the direct vasodilatory effect of arotinolol and the underlying mechanisms. Moreover, we also comprehensively investigated whether arotinolol had any effect on vascular stiffness and the corresponding changes in endothelial function, arterial stiffness and vascular wall composition in SHR, due to few reports in vessel protection by arotinolol.

## Materials and Methods

### Animals and experimental groups

Male SHRs and WKY rats, 16-week-old were purchased from the Shanghai Experimental Animal Center. SHRs were randomly divided into 3 groups, and treated with vehicle (SHR control, n = 12), metoprolol (200 mg·kg^−1^·day^−1^, n = 12), or arotinolol (30 mg·kg^−1^·day^−1^, n = 12) for eight weeks respectively. Age-matched WKY (n = 12) was used as normotensive control. Meanwhile, another 24 16-week-old WKY rats were used in the measurement of isometric force to elucidate the vasodilating effects of arotinolol and underlying mechanisms. All rats were housed in a reversed 12∶12-hour light-dark cycle in a temperature-controlled facility (21±1°C) with free access to standard laboratory chow and tap water. SHRs in the metoprolol and arotinolol groups were treated with drugs in 0.5 ml water by oral gavage with a feeding needle twice every day for 8 weeks, while WKY group and another control group of SHRs received only 0.5 ml regular water by oral gavage for 8 weeks. Animals were anesthetized via a 60 mg/kg body weight intraperitoneal injection of pentobarbital sodium and then killed by overdose of pentobarbital sodium. The investigation conformed to National Institutes of Health Guidelines for the Care and Use of Laboratory Animals, and was approved by the ethics review board of animal experiments at Ruijin Hospital.

### Vessel preparation and measurement of isometric force in rat vascular rings

Rat thoracic aortas, renal arteries, and mesenteric arteries were dissected, cleared of fat and connective tissues, cut into 2–3 mm rings, and mounted on isometric force transducers (Danish Myo Technology Model 610 M, Denmark) in a 5-mL organ bath, and aerated with 95% O_2_ and 5% CO_2_ under an initial resting tension of 19.6 mN, 2.5 mN, and 1 mN, respectively. Force was recorded via a PowerLab/8sp data acquisition system (A.D. Instruments, Castle Hill, Australia). Acetylcholine-induced vasodilations over 80% were regarded as endothelium-intact vessels, whereas lack of relaxation by acetylcholine was taken as endothelium removal.

To evaluate the vasodilatory effect of arotinolol and metoprolol, cumulative concentration-response curves of arotinolol and metoprolol (10^−8^–10^−5^ mol/L) were constructed in three different vascular rings pre-contracted with 1 µmol/L phenylephrine. To evaluate the underlying mechanisms of vasodilations by arotinolol, in some groups, aortic rings were incubated in advanced with N^ω^-nitro-l-arginine methyl ester (l-NAME, 10^−4^ mol/L), tetraethylammonium (TEA, 10^−2^ mol/L, a potassium channel inhibitor), glibenclamide (10^−4^ mol/L, an ATP-sensitive potassium channel inhibitor), or 4-aminopyridine (2.5×10^−3^ mol/L, a voltage-gated potassium channel inhibitor) for 30 min before addition of phenylephrine,then cumulative concentration-response curves of arotinolol (10^−8^–10^−5^ mol/L) were constructed. To evaluate the beneficial effect of metoprolol and arotinolol on endothelial function, the vasodilatory response to acetylcholine was evaluated in aortas from WKY, SHR control, and SHR rats treated with metoprolol and arotinolol after 8 weeks' treatment.

### Measurement of tail systolic blood pressure, central arterial pressure and pulse wave velocity

Tail systolic blood pressures (SBP) were measured at the beginning of experiment and at the end of week 8 in conscious rats by Softron tail-cuff BP-98A non-invasive sphygmomanometer (Softron Incorporated, Tokyo, Japan).

Central arterial pressure (CAP) and pulse wave velocity (PWV) were measured after 8 weeks' treatment as previously described. [10] Briefly, in order to determine the propagation timing, 2-Fr Mikro-tip catheter pressure transducers (Model SPR-407; Millar Instruments, Houston, TX) were implanted into the aortic arch, and the abdominal aortas proximal to the iliac bifurcation. Then two Millar catheters were connected to an amplifier (Powerlab ML118/D; A.D. Instruments) and a PowerLab/8sp data acquisition system at a sampling rate of 1000 Hz to generate the time difference between the diastolic phase centers of the proximal and distal waves. A total of five–nine cycles were averaged as one propagation time. Then, PWV was calculated by dividing the propagation distance between two catheter tips by the propagation time. The central arterial pressure, which represented the systolic pressure of aortic root, was measured by the proximal pressure transducer located in the aortic arch.

### Intracellular NO and Ser^1177^ phosphorylation of endothelial nitric oxide synthase (p-eNOS) assays in human aortic endothelial cells

Human aortic endothelial cells (HAEC) were purchased from BioLeaf (Shanghai BioLeaf Biotech Co., Ltd, Shanghai, China) and cultured in the medium at 37°C under 5% CO_2_.[11] Cells were made quiescent by incubation in serum-free culture medium for 24 h before examination.

After pretreatment under serum-free conditions with 4-amino-5-methylamino-2′7′-difluorofluorescein diacetate (DAF-FM; final concentration, 5 µmol/L; excitation wavelength, 495 nm, emission wavelength, 515 nm; Molecular Probes, Eugene, OR, USA) for 30 min at 37°C and 5% CO_2_, and rinsing with PBS, HAECs were incubated with 10 µmol/L arotinolol or metoprolol. Next, the fluorescence intensity of NO was measured using Varioksan Flash fluorescent microplate readers (Thermo Electron Corporation, USA).

To clarify whether arotinolol cause Ser1177 phosphorylation of eNOS, another part of HEACs lysates, which were pretreated with 10 umol/l arotinolol for 2 5 15 30 60 minutes, were subjected to SDS-PAGE gel electrophoresis and electrophoretically transferred to a nitrocellulose membrane as described previously. The membranes were then exposed to primary antibodies of p-eNOS (1∶500 dilution; Enzo Life Sciences, Switzerland) or anti-eNOS antibody (1∶500 dilution; Enzo Life Sciences, Switzerland) overnight at 4°C. After incubation with the peroxidase-linked secondary antibody for 1 hour at room temperature, immunoreactive proteins were visualized by Western Lightning plus ECL (Perkin Elmer, Boston, MA, USA), and finally exposed to X-ray films for the detection of target proteins.

### Western blotting in the aortas of rats

Phosphorylation levels of endothelial nitric oxide synthase (eNOS) protein were evaluated. [12] Briefly, soluble protein extracts (20 µg) from the homogenized aortas were loaded into 8% SDS-polyacrylamide gels and transferred to PVDF membranes. After blocking in non-fat milk, the membranes were exposed to polyclonal rabbit anti-Ser1177 phosphorylation eNOS antibody (1∶500 dilution; Abcam Laboratories, UK) or polyclonal rabbit anti-eNOS antibody (1∶500 dilution; Enzo Life Sciences, Switzerland) overnight at 4°C. After incubation with HRP-linked secondary antibodies, the membranes were visualized by enhanced chemiluminescence reagent, and finally exposed to x-ray films for the detection of target proteins.

### Collagen content and immunohistochemistry

After recording body weights, the thoracic aortas were dissected from rats and subjected to hydrolysis in HCl for 16 h. Next, hydroxyproline content was evaluated by the chloramine T and paradimethylaminobenzaldehyde method. Collagen content was calculated as (hydroxyproline content) ×7.46. [13]

Immunohistochemistry of the aortic tissues was performed as described previously. [14] Sections were incubated with commercial rabbit polyclonal antibodies against collagen type I (Abcam Laboratories, UK) at 1/1000 dilution overnight at 4°C, and conjugated with horseradish peroxidase (HRP) antibody (1∶500 dilution; Santa Cruz Biotechnology, Santa Cruz, CA) at room temperature. Subsequently, all fields were photographed using a Nikon E600 light microscope (Nikon, Tokyo, Japan) at ×200 magnification.

### Drugs

Drugs used were phenylephrine hydrochloride, acetylcholine chloride, N^ω^-nitro-l-arginine methyl ester (l-NAME), tetraethylammonium (TEA), glibenclamide, and 4-aminopyridine (4-AP) (Sigma-Aldrich, St. Louis, Missouri, USA).

Arotinolol and metoprolol were provided by Sumitomo Pharma Co., Ltd.

### Statistical analysis

Vasodilatory effects were expressed as percentage tension of phenylephrine-induced preconstriction. Results are expressed as the mean ± SEM values. Comparisons were made by using Student's t-test or one way ANOVA analysis with Bonferroni test, if more than two groups were compared. Statistical significance was indicated by P<0.05.

## Results

### Vasodilatory effects of arotinolol and metoprolol in rat aortas, renal arteries and mesenteric arteries

**Figure 1:**
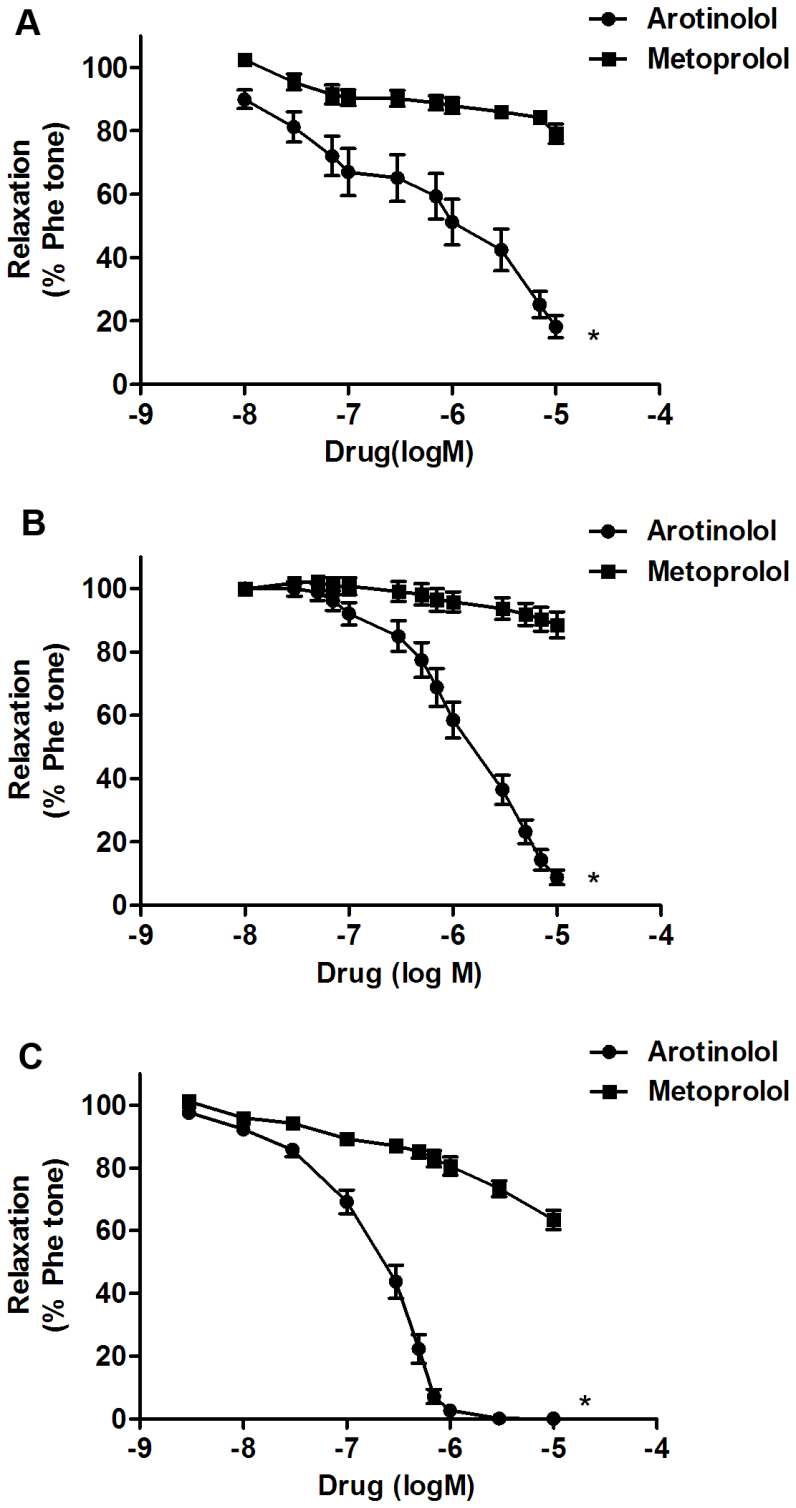
Vasodilations in three various arteries. Vasodilatory effects of arotinolol and metoprolol in rats' aortas (A), renal (B) and mesenteric arteries (C). ^*^ *P*<0.05 versus metoprolol. Data are expressed as percentage tension of phenylephrine-induced preconstruction. n = 8 in each group.

To determine vasodilatory effects of arotinolol and metoprolol, vasodilations of vascular rings were evaluated by isometric force recording. As shown in Fig. 1, arotinolol dose-dependently relaxed endothelium-intact vascular rings from rat aortas, renal arteries and mesenteric arteries; whereas metoprolol had few vasodilatory effects in the three various vascular rings. Notably compared with the aortas, the vasodilator response curves in the mesenteric arteries dropped more sharply, indicating that the vasodilatory effects of arotinolol were more obvious in mesenteric arteries than in aortas.

### Arotinolol-induced vasodilation involves NO and K^+^ channels

**Figure 2:**
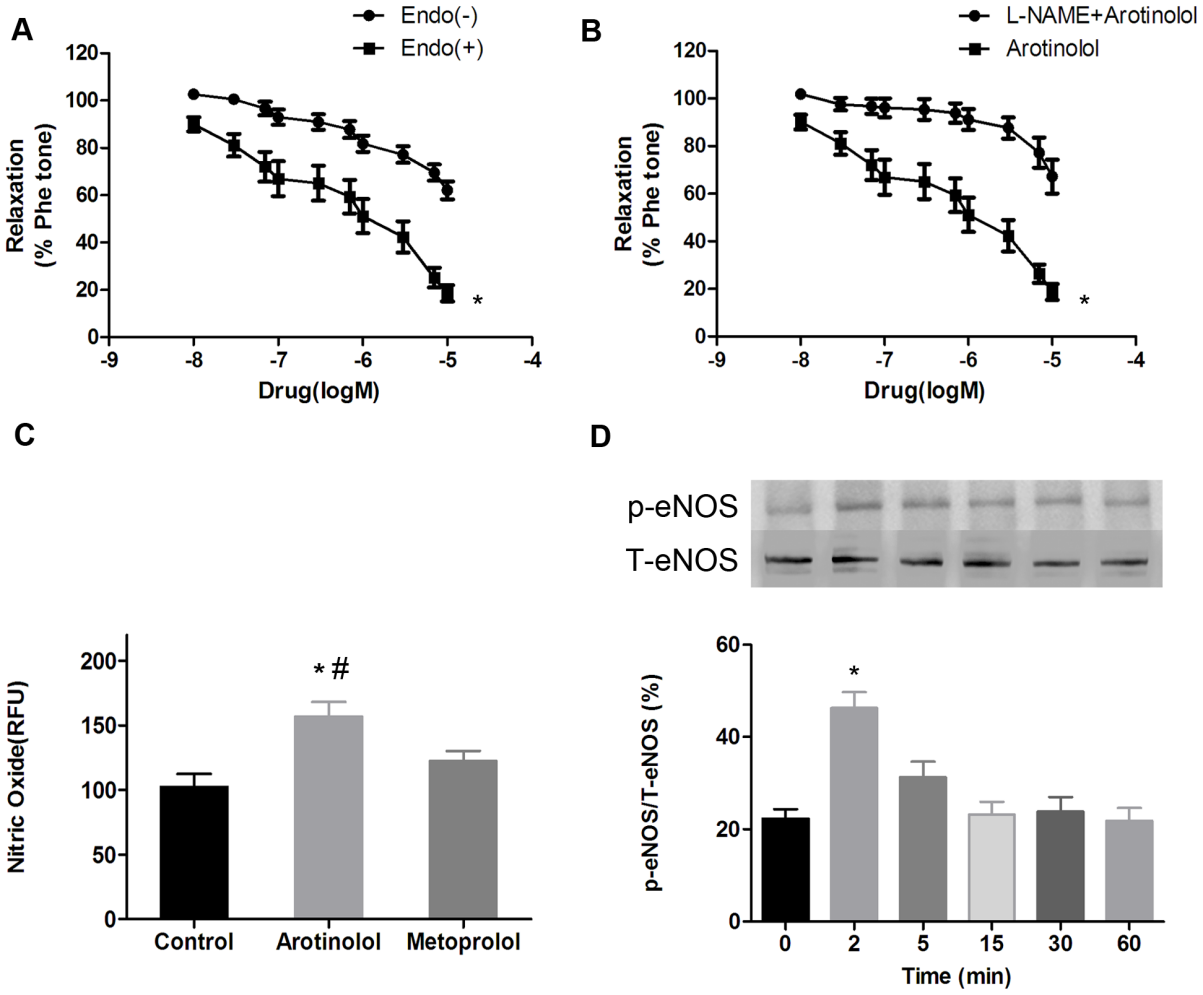
Vasodilations by arotinolol involve endothelium-derived NO. (A) Cumulative concentration-response curves of arotinolol and metoprolol in thoracic aortas with or without endothelium. ^*^ *P*<0.05 for with vs. without endothelium. (B) Cumulative concentration-response curves of arotinolol in the presence or absence of L-NAME in the rat aortas. ^*^ *P*<0.05 for arotinolol vs. arotinolol + L-NAME. n = 8 in each group. (C) Effects of different β-blockers on NO production in HAECs. ^*^ *P*<0.05 for arotinolol versus control. ^#^ *P*<0.05 for arotinolol vs. metoprolol groups. (D) Effect of arotinolol on p-eNOS/T-eNOS ratios by western blotting assay in HAECs. ^*^ *P*<0.05 for 2 minutes group vs. control group (0 minute group). n = 12 in each group.

The vasodilatory mechanism of arotinolol was then investigated. In endothelium-denuded rings, arotinolol produced less relaxation (Fig. 2A). Similarly, pretreatment of endothelium-intact rings with eNOS inhibitor L-NAME (100 µmol/L) effectively reduced arotinolol-induced relaxations (Fig. 2B). These results indicate that endothelium-derived NO was involved in arotinolol-induced vasodilation.

The effects of arotinolol on NO production and eNOS phosphorylation were then evaluated since it represented the most important regulatory mechanism in eNOS function. As shown in Figure 2C&D, NO production was significantly increased after HAECs were treated with arotinolol for 30 min. However, this phenomenon did not occur in metoprolol pretreatment. Furthermore, p-eNOS/T-eNOS ratio also went up obviously when HAECs being treated with arotinolol in 2 min. These results indicate that arotinolol-induced vasodilation involves eNOS phosphorylation.

**Figure 3:**
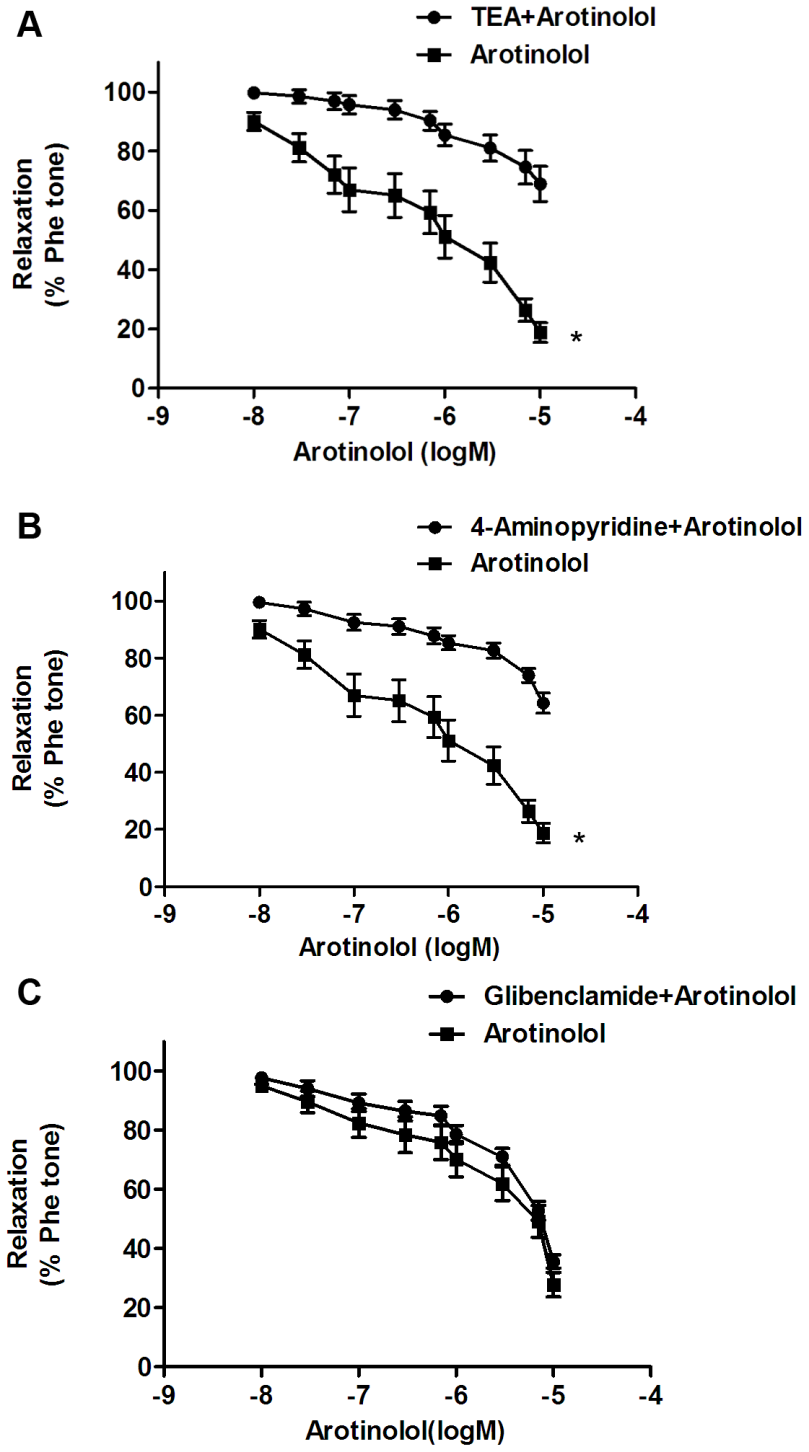
Involvement of potassium channels. (A) Cumulative concentration-response curves to arotinolol in the presence or absence of potassium channel inhibitor (TEA). ^*^ *P*<0.05 for arotinolol vs. arotinolol + TEA. (B) Cumulative concentration-response curves to arotinolol in the presence or absence of 4-aminopyridine. ^*^ *P*<0.05 for arotinolol vs. arotinolol + 4-aminopyridine. (C) Cumulative concentration-response curves to arotinolol in the presence or absence of glibenclamide. *P*>0.05 for arotinolol vs. arotinolol + glibenclamide. n = 8 in each group.

The possible involvement of large conductance Ca^2+^-activated K+ (BK_Ca_) channels, voltage-gated K+ (Kv) channels, and ATP-sensitive K+ (K_ATP_) channels in arotinolol-induced vasodilation was also investigated, since these three channels was closely related to NO-induced vasodilation. As shown in Fig. 3, arotinolol-induced relaxations were inhibited by BK_Ca_ channels inhibitor tetraethylammonium ions (TEA+, 1 mmol/L) and Kv channels inhibitor 4-aminopyridine (2.5×10^−3^ mol/L), but K_ATP_ inhibitor-glibenclamide did not inhibit the vasodilation by arotinolol. These results indicate that arotinolol relaxed rat aortic rings partially through Kv channels.

### Effects of arotinolol on tail SBP, CAP and PWV in SHRs

To evaluate the effects of chronic treatment of arotinolol and metoprolol on blood pressure, SHR were treated with arotinolol and metoprolol for 8 weeks and the changes of tail SBP and CAP were then detected.

**Figure 4:**
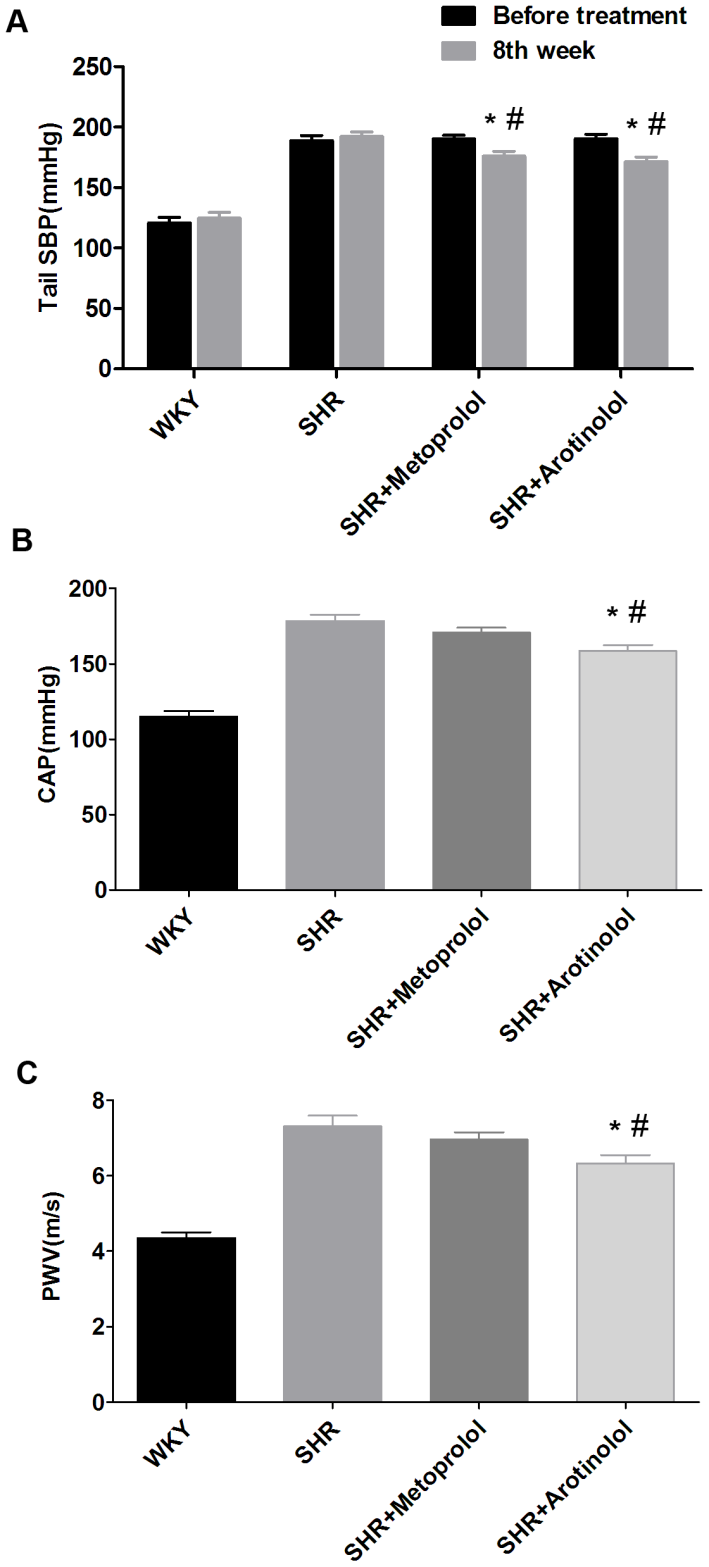
Tail SBP, CAP and PWV in SHRs treated with arotinolol and metoprolol. (A) Tail SBP before treatment and at week 8 in WKY, SHR control, or SHR treated with metoprolol or arotinolol. *P<0.05 vs. those origins before treatment, ^#^ *P*<0.05 vs. SHR control at week 8, *P*>0.05 for arotinolol vs. metoprolol groups at week 8. Central aortic pressure (B) and Pulse wave velocity (C) in WKY, SHR control, or SHR treated with metoprolol or arotinolol. *P<0.05 vs. SHR control, ^#^ *P*<0.05 vs. SHR treated with metoprolol. n = 12 in each group.

As depicted in Fig. 4A, tail SBP of arotinolol and metoprolol groups at week 8 dropped markedly compared with those origins before treatment and those of untreated SHR at week 8, while no differences were observed in tail SBP between arotinolol and metoprolol groups at week 8. Nevertheless, CAP was significantly higher in SHR than that in WKY. Treatment with arotinolol decreased CAP, while metoprolol did not work with the similar drop in tail SBP between arotinolol and metoprolol group at week 8 (Fig. 4B).

Meanwhile, PWV was detected to evaluate the effects of arotinolol and metoprolol on stiffness of large artery after arterial pressures were measured above. The results showed PWV were sharply higher in SHR than that in WKY as well, indicating that stiffness of large arteries was increased in SHR. Moreover, PWV of arotinolol group obviously dropped, compared with those of metoprolol group at week 8 (Fig. 4C).

These results indicate that arotinolol, but not metoprolol, decreased large artery stiffness in SHR.

### Arotinolol improved endothelial function and eNOS phsophorylation in thoracic aortas in SHRs

**Figure 5:**
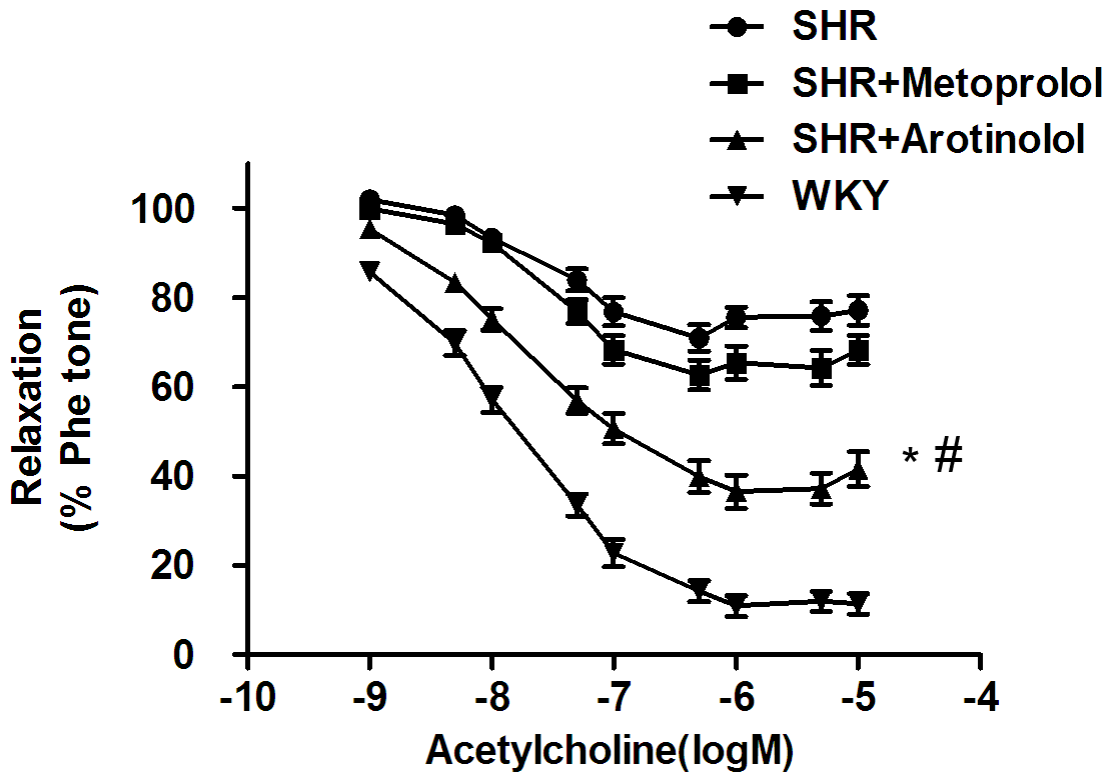
Effects of arotinolol and metoprolol on endothelium-dependent vasodilation in thoracic aortas. Cumulative concentration-response curves to acetylcholine in phenylephrine- precontracted aortic rings in WKY, SHR control, or SHR treated with arotinolol or metoprolol. ^*^ *P*<0.05 vs. SHR control. ^#^ *P*<0.05 versus SHR treated with metoprolol. n = 12 in each group.

The effects of arotinolol and metoprolol on endothelial function were also investigated since endothelial functions are involved in arterial stiffness. Furthermore, endothelial dysfunction usually is characterized by impaired endothelium-dependent vasodilation to acetylcholine. Fig. 5 showed that the vasodilatory responses to acetylcholine were significantly decreased in SHRs compared with normotensive WKY rats, indicating that endothelial function was impaired in SHR. Arotinolol increased vasodilatory response to acetylcholine, while metoprolol had no effect. These results suggest that arotinolol, but not metoprolol, improved endothelial function in SHR.

**Figure 6:**
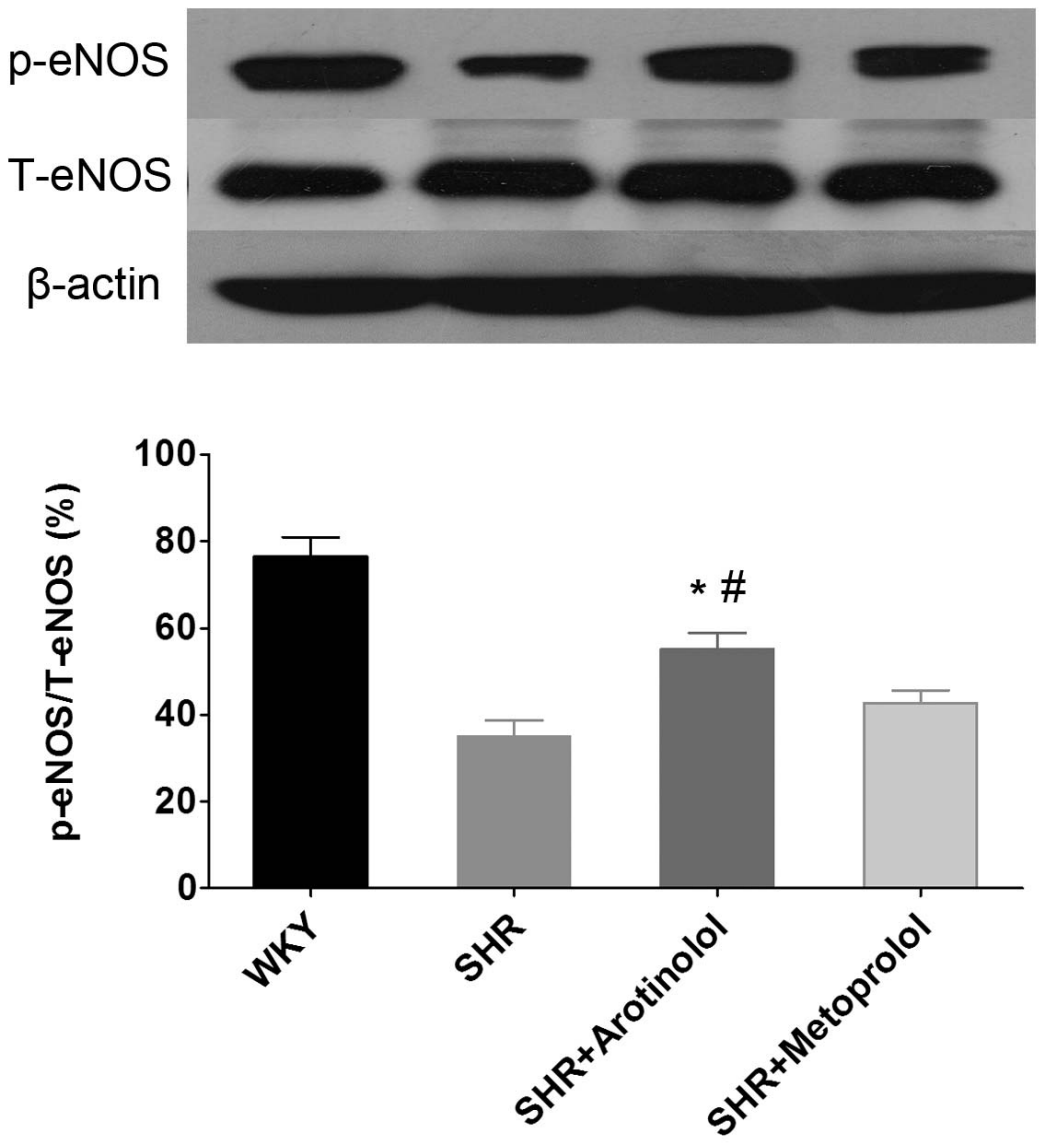
Ser1177 phosphorylation of eNOS in thoracic aortas in SHRs. Effects of metoprolol or arotinolol on aortic p-eNOS level in SHR by western blotting assay. *P<0.05 vs. SHR control, ^#^ *P*<0.05 vs. metoprolol-treated group. n = 12 in each group.

Next, we examined whether arotinolol-induced vasodilation improvement involved eNOS phosphorylation in the rat aorta. As shown in Fig. 6, SHR showed a remarkable reduction in p-eNOS compared with the WKY rat as shown in the decreased p-eNOS/T-eNOS ratio, indicating that endothelial function was impaired in SHR. After treatment with arotinolol, p-eNOS levels were significantly increased in the SHR-rats aortas compared with SHR controls or SHRs treated with metoprolol. These results indicate that arotinolol improved endothelial dysfunction through increasing eNOS phosphorylation.

### Arotinolol decreased collagen content in rat aortas

**Figure 7:**
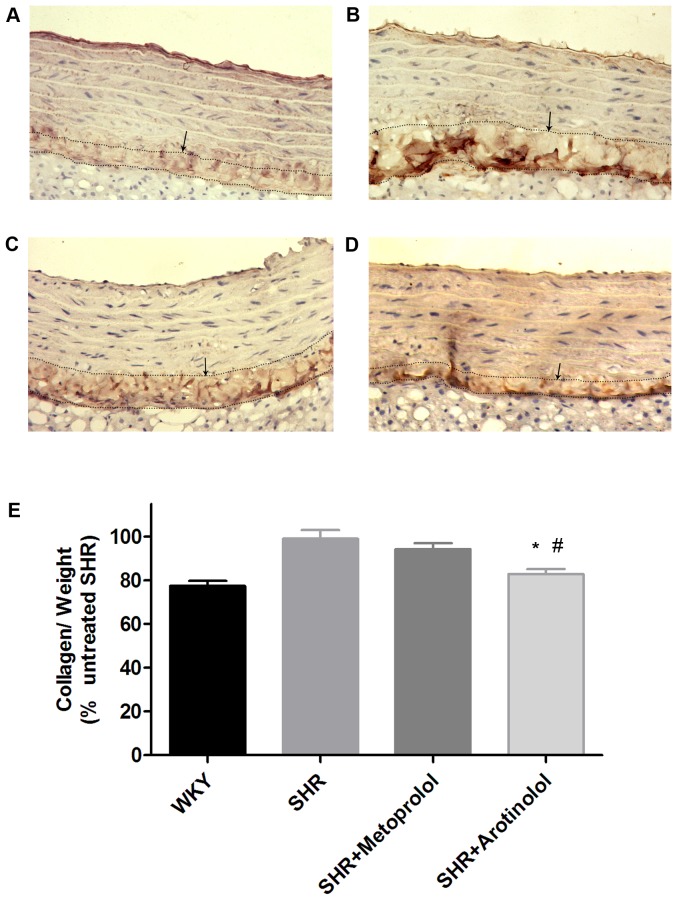
Collagen contents in rat aortas by immunohistochemistry and biochemistry assay. Immunohistochemistry of collagen I, which was mainly distributed in the adventitia of rat aortas (×200). (A)WKY, (B)SHR control, (C)SHR treated with Metoprolol, (D) SHR treated with Arotinolol. (E) Summarized data showing the changes in collagen contents of aortas in WKY, SHR control, SHRs treated with metoprolol or arotinolo by chloramine T and paradimethylaminobenzaldehyde method. *P<0.05 vs. SHR control, ^#^ *P*<0.05 vs. SHR treated with metoprolol. n = 12 in each group.

We then determined whether arotinolol and metoprolol had any effects on collagen I deposition in rat aortas, since it has been well established that collagen content played a critical role in arterial stiffness of large arteries. As shown in Fig. 7, the expression of collagen I was higher in SHR compared with age-matched WKY in immunohistochemistry. Further, biochemical assay showed that collagen content was higher in SHR compared with age-matched WKY. These results indicate the increased collagen deposition in thoracic aorta in SHR, which may be associated with increased arterial stiffness of thoracic aorta. Moreover, as shown in Fig. 7, arotinolol significantly decreased collagen content in both immunohistochemistry and collagen content assay, whereas metoprolol did not happen. These results indicate that arotinolol decreased abnormal collagen deposition in thoracic aortas, which may be related to its beneficial effect on vascular stiffness.

## Discussion

The present study demonstrates that arotinolol-induced vasodilation involves eNOS phosphorylation, NO production and Kv channels. Arotinolol effectively decreased arterial stiffness in SHRs as indicated by decreased CAP and PWV, which was accompanied by the increased eNOS phosphorylation/NO production, and decreased collagen deposition in SHRs. Meanwhile, metoprolol takes no effects on vascular protection. Our investigation suggests that the beneficial effect of arotinolol on arterial stiffness may involve the direct influences in endothelial NO in SHRs.

In the present study, arotinolol induced obvious relaxant effects on the three kinds of arteries, while metoprolol did not. Arotinolol-induced vasorelaxations were largely inhibited by the eNOS inhibitor L-NAME, and aroninolol significantly increased the eNOS phosphorylation, suggesting that endothelium-derived NO mediates arotinolol-induced relaxations. Furthermore, this study shows that arotinolol increased p-eNOS (Ser1177) level in cultured endothelial cells. These results indicate that arotinolol-induced vasodilation involves enhanced eNOS phosphorlation and increased NO production. This finding is novel since it demonstrates for the first time that arotinolol has direct vasodilatory effect via NO pathway. Moreover, we found that Kv channels were also involved in arotinolol-induced vasodilation, while voltage-gated potassium channels are reportedly involved in the pathogenesis of hypertension, and thus, can be useful as novel therapeutic targets. [15], [16]

Meanwhile as known to all, NO plays a key role in the aetiology and development of hypertension, and recent researches show that potassium channel dysfunction is one of the determinants to induce arterial hypertension.[17], [18], [19] This study suggests that arotinolol exactly acts on both targets. It can either enhance Ser1177 phosphorylation of eNOS to release NO in HAECs, or cause vasodilation by affecting potassium channel. Therefore, arotinolol likely has a significant effect on the treatment of hypertension by a novel pathway.

Some studies indicate that arterial stiffness has increasingly become an important prognostic index and potential therapeutic target in hypertension and PWV is a well-recognized classic index for assessing arterial stiffness. [20], [21] In the present study, we showed that metoprolol had no effect on arterial stiffness since there was no significant difference in PWV and CAP in metoprolol-treated SHR compared with SHR control, although it caused the similar drop in tail SBP compared with arotinolol-treated SHR, which is consistent with previous studies showing that metoprolol had no influence in arterial stiffness in human and animal hypertensive objects. Nevertheless, arotinolol significantly decreased arterial stiffness as shown in the decreased PWV and CAP in arotinolol-treated SHR compared with SHR control. These findings suggest that arotinolol plays a protective role against arterial stiffness.

It has been demonstrated that the composition and properties of vascular walls, such as collagen content, [22]endothelial dysfunction, [9]endothelial nitric oxide synthase (eNOS), [23]and NO levels, [24] all play important roles in the development of vascular stiffness and hypertension. Endothelial dysfunction, usually characterized by impaired endothelium-dependent vasodilation in response to acetylcholine, plays a key role in the development of hypertension and its complications, [9] [25] Furthermore, Wallace SM et al has described that aortic PWV was associated with endothelial dysfunction. [26] Consistent with previous studies,[9] the present study shows that endothelium-dependent vasodilation was significantly lower in SHR compared with age-matched WKY, which might be caused by the lower eNOS phosphorylation level in thoracic aorta in SHR as shown in the present study. Furthermore, the present study shows that arotinolol, but not metoprolol, significantly improved endothelial dysfunction as illustrated in increased vasodilation in response to acetylcholine stimulation. Consistent with the improved vasodilation, the present study also shows that arotinolol, but not metoprolol increased eNOS phosphorylation in SHR. These results indicate that arotinolol had a better effect on improvement of endothelium-dependent vasodilations than metoprolol.

Collagen is the key determinant of arterial stiffness. Barnes MJ et al observed that type I collagen is believed to impart arterial stiffness.[22] It has been shown that the total aortic collagen content or aortic fibrosis of the untreated SHR was considerably higher than that of the normotensive WKY rats. [27], [28] Our investigation illustrated that arotinolol decreased aortic collagen contents in the SHR, indicating that arotinolol improved arterial stiffness at least partially by reducing aortic collagen deposition.

In vivo experiments of this study it indicates that arotinolol can reduce PWV and CAP which reflect arterial stiffness by improving endothelium-dependent vasodilations and collagen contents in the SHR aortas. Moreover, some studies present PWV as an independent risk factor of cardiovascular event, closely related to cardiovascular mortality.[29], [30] Arterial stiffening per se is the cause of the poor response of blood pressure to chronic drug treatment.[31] In the present study, arotinolol is shown to obviously reduce arterial stiffness in SHRs. Therefore arotinolol would be beneficial to the treatment of hypertension and its complications as a de-stiffening antihypertensive medication. Further studies should be designed to verify vascular function for arotinolol in a clinical trial.

In conclusion, our results suggest that arotinolol has direct vascular protective effect by increasing eNOS phosphorylation and NO production both in vivo and in vitro. Furthermore, arotinolol decreased arterial stiffness and aortic collagen content in SHR. The effect on vessel protection, in combination with the α-adrenergic receptor blocking effect, indicates that arotinolol is a superior β-blocker compared with other β-blockers. Few data is available at present with regards to NO production of other α/β-blockers, thus additional research is required for further study.
